# Combination of Fractional Exhaled Nitric Oxide (FeNO) Level and Asthma Control Test (ACT) in Detecting GINA-Defined Asthma Control in Treated Asthmatic Patients in Vietnam

**DOI:** 10.1155/2020/5735128

**Published:** 2020-04-25

**Authors:** Vinh Nguyen Nhu, Pham Le An, Niels H. Chavannes

**Affiliations:** ^1^Department of Public Health and Primary Care, Leiden University Medical Center, Leiden, Netherlands; ^2^Department of Family Medicine, Faculty of Medicine, University of Medicine and Pharmacy at Ho Chi Minh City, Vietnam; ^3^Department of Respiratory Functional Exploration, University Medical Center, Ho Chi Minh City, Vietnam

## Abstract

**Background:**

FeNO has been used as a marker for Th2-mediated airway inflammation in asthma. There is evidence which recommends the use of this biomarker in asthma management. Little is known about whether the FeNO test alone or in combination with the ACT score can reflect asthma control in Vietnamese patients.

**Materials and Methods:**

A cross-sectional study was conducted in asthmatic patients (≥18 years old) recruited at the University Medical Center, Ho Chi Minh City, Vietnam from March 2016 to March 2017. Asthma control levels were assessed following the GINA 2017 guidelines, and FeNO was measured by a Niox Mino device. FeNO cut-offs predicting asthma control status were determined using the ROC curve analysis. The combination of FeNO and ACT was investigated in detecting well-controlled and uncontrolled asthma. The results of the study are as follows: 278 patients with 68% females, mean age of 44 years, and mean asthma duration of 10 years were analyzed. All patients were treated following step 2 to 4 of GINA guidelines. Mean (SD) FeNO was 30.6 (24) ppb. Patients with uncontrolled (16%), partly controlled (29%), and well-controlled asthma (55%) had a median (IQR) FeNO of 50.0 (74), 25.0 (23), and 21.0 (22.3) ppb, respectively, and the mean of FeNO in the uncontrolled group was significantly higher than that in other groups (*p* < 0.001). The area under the ROC curve (AUC) for FeNO detecting uncontrolled asthma was 0.730 with an optimal cut-off point of FeNO > 50 ppb, and this AUC increased to 0.89 when combining FeNO and ACT. The AUC for FeNO detecting well-controlled asthma was 0.601 with an optimal cut-off point of FeNO <25 ppb and this AUC increased to 0.78 if combining FeNO and ACT.

**Conclusions:**

FeNO can predict asthma control status with an estimated cut-off point of <25 ppb for well-controlled and >50 ppb for uncontrolled asthma. The combination of FeNO and ACT provides better information regarding asthma control than FeNO alone, and this combination is useful to predict asthma control statuses in asthmatic patients in Viet Nam.

## 1. Introduction

Recent asthma guidelines have recommended using a control-based strategy to manage asthma patients with the goal of helping them achieve and maintain disease control, which requires the suppression of airway inflammation by inhaled corticosteroids (ICS) or other medications [1]. In addition, although published evidence is still controversial, many clinical trials were conducted and indicated that the strategy of asthma management based on or combined with the FeNO test could provide better outcomes than based on symptoms and lung function only [2–4]. In Vietnam, the FeNO test has been developed recently, and this biomarker is emerging to be used in asthma management in this country. However, whether this biomarker reflects asthma control in Vietnam is still not yet determined although the literature reported that it was a weak correlation between FeNO and asthma control level [5]. Therefore, before FeNO being used widely in Vietnam for asthma management, its value (alone or combination with other parameters) in reflecting asthma control should be investigated. Thus, the aim of this study is to determine the value of FeNO alone or combined with ACT in reflecting GINA-defined asthma control.

## 2. Patients and Methods

### 2.1. Study Design and Setting

This was a prospective cross-sectional study. Eligible participants were ambulatory adult patients (≥18 years old) (recruited between March 2016 and March 2017) at the University Medical Center Asthma and COPD clinic, Ho Chi Minh City, Vietnam.

### 2.2. Ethical Approval

The study protocol was approved by the Institutional Review Board of the University of Medicine and Pharmacy, Ho Chi Minh City, Vietnam. All patients were given a written informed consent form, and those who participated in this study had to sign this consent form.

### 2.3. Inclusion and Exclusion Criteria

Patients eligible for inclusion in this study were ≥18 years old, diagnosed with asthma at least 6 months previously according to the GINA 2015 criteria (had asthma symptoms and evidence of bronchodilator reversibility test—FEV1 change ≥ 12% and 200 ml after inhalation of 4 puffs (400 mcg) salbutamol—of Ventolin®), and followed up at this clinic. Patients were excluded if meeting any of the following exclusion criteria: hospitalized for asthma or had an acute upper or lower respiratory tract infection within 4 weeks prior to the study, had a known respiratory disorder other than asthma and/or systemic/thoracic abnormalities that influence normal lung function, current smoker or ex-smoker with >10 pack-years, and did not undergo their treatment for more than 2 weeks within 3 months prior to the study.

### 2.4. Sample Size and Sampling Technique

With a presumption based on the literature that FeNO can detect GINA-defined uncontrolled (UC) asthma with a specificity of 80% [6], the calculated sample size was 246 subjects.

### 2.5. Data Collection and Classification

- Asthma control is classified into the following 3 levels: well-controlled (WC), partly controlled (PC), and uncontrolled (UC) based on GINA 2017 guidelines as given in Table 1.
- FeNO measurement: FeNO level was measured by a Niox Mino device (Aerocrine AB, Solna, Sweden) at a flow rate of 50 mL/s for 10 seconds following the manufacturer's manual [7, 8]. FeNO measurement was followed to the ATS/European Respiratory Society (ERS) 2005 recommendations [9]. Participants indicated for FeNO measurement underwent this test prior to a spirometry test to avoid interfering the spirometry results. FeNO values were graded into the following 3 levels: low (<25 ppb), intermediate (25–50 ppb), and high (>50 ppb) following the American Thoracic Society (ATS) categorization [10].
- Asthma Control Test (ACT): This test comprises five questions assessing the frequency of shortness of breath, frequency of asthma night-time symptoms, degree of functional limitation, frequency of using rescuers, and patient's self-assessment of their level of asthma control. Each item has five response choices (each with a score ranging from 1–5). Accordingly, the levels of asthma control are as follows: well-controlled (scores of 20–25), partially controlled (15–19), and uncontrolled (5–14) [27]. The Vietnamese version of this questionnaire was validated [28] and used for the present study.
- Spirometry: The spirometry test was conducted by using a KoKo spirometer (nSpire Health, Longmont CO 80501, USA) following the manufacturer's instructions [11]. Devices' calibration and patients' preparation before the measurement were conducted following the guidance of ERS/ATS 2005 [12]. Participants performed spirometry after FeNO measurement. Spirometry variables used in this study comprise of FEV1 % predicted (%FEV1) and PEF% predicted (%PEF), which are commonly used in asthma monitoring.

### 2.6. Data Analysis

Data were processed with Epidata software and analyzed using SPSS software. Continuous variables are presented depending on their distribution as means and standard deviations (SD) for normal distribution or median and interquartile range (IQR) for nonnormal distribution. Ordinal and nominal variables are presented as numbers and percentages. The chi-square statistic was used for testing relationships between categorical variables. Student's *t*-test and ANOVA test were used to compare the means of two groups or more, respectively, for normally distributed data (such as FEV1). The Mann–Whitney *U* test and the Kruskal–Wallis test were used to compare the median of two groups or more, respectively, for nonnormally distributed data (such as FeNO). If a comparison of multiple groups provides significant difference, the post hoc analysis then was used for pair-wise comparison of post hoc ANOVA with the Tamhane test—because of unequal variances—for parametric test and the Mann–Whitney *U* test for nonparametric test. The receiver operating curve (ROC) analysis was used to distinguish WC asthma and UC asthma by FeNO levels or a combination of FeNO and ACT as well as identify cut-off points with an optimal sensitivity (true-positive rate) and specificity (true-negative rate) using the highest Youden index. Positive predictive value (PPV), negative predictive value (NPV), positive likelihood ratio (PLR), negative likelihood ratio (NLR), and accuracy of the test were also calculated. A *p* value ≤0.05 was considered statistically significant.

## 3. Results

### 3.1. Characteristics of Study Patients

**Figure 1:**
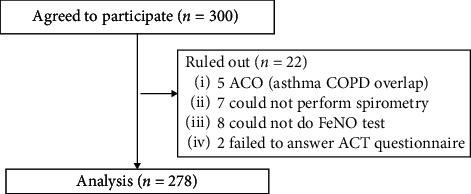
Flowchart of number of patients recruited and analyzed in this study.

A number of patients were recruited and analyzed as shown in the flowchart (Figure 1).

**Figure 2:**
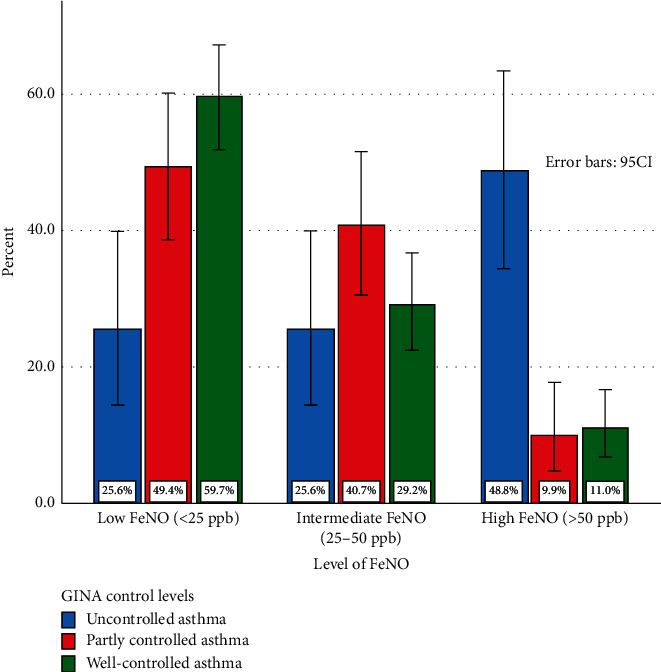
The distribution of patients in different levels of asthma control according to the FeNO level classification following the ATS categorization, *p* < 0.001.

**Figure 3:**
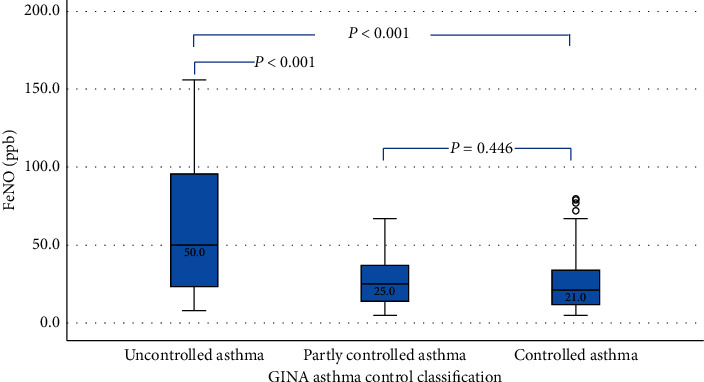
The median of fractional exhaled nitric oxide (FeNO) levels in patients with different asthma control status according to the GINA guidelines.

Totally, 278 eligible subjects were inputted into the study analysis after 300 patients being invited (93%). The demographic data of the study subjects are given in Table 2. In the present study, almost all patients received ICS with or without combining with long-acting beta2-agonists (LABA). Most of the subjects were classified as WC group (55%). The present study have objectives to detect GINA-defined asthma control (UC and WC); thus, comparisons of many characteristics of the study participants according to the levels of GINA asthma control are presented in Tables 2 and 3 and Figures 2 and 3. The shortest and longest durations of asthma were 6 months and 73 years, respectively. Thirty-nine patients (14%) reported acquiring asthma from childhood. The mean (SD) and median (IQR) FeNO level of the study subjects were 30.6 ppb (24) and 24 ppb (26.3), respectively.

The prevalence of patients with FeNO corresponding to three levels following the American Thoracic Society (ATS) categorization [10] were 51% (low), 32% (intermediate), and 17% (high), respectively. The distribution of patients with different levels of asthma control according to FeNO levels is shown in Figure 2. There are only 31 (11%) of patients with WC asthma but nearly a half of the UC asthmatic patients (48.8%) had high FeNO levels. Most of the WC patients with asthma (59.7%) and one-fourth (25.6%) of the UC patients with asthma had low FeNO levels. On the other hand, most patients (46%) with high FeNO level had UC asthma, while most patients (64%) with low FeNO level had WC asthma.

Levels of asthma control may be contributed by many factors in which severity of the disease and its treatment play an important role. FEV1 % predicted (%FEV1) is still a component of asthma severity, and the distribution of this parameter in different asthma control levels is given in Table 3. 16.3% UC asthma patients had %FEV1 less than 60% and that of the percentage in the PC and WC groups are quite low (7.4% and 1.3%). Most WC patients (83, 8%) had normal %FEV1 (%FEV1≥80%).

Regarding asthma treatment, the prevalence of patients who were on GINA treatment steps 1, 2, 3, 4, and 5 were 0%, 22.3%, 31.2%, 45.7%, and 0%, respectively. Table 3 shows different percentage of asthma treatment levels according to asthma control status. Most of the UC patients (67.4%) were treated with GINA treatment step 4, and many patients (32.5%) with WC asthma were treated with GINA treatment step 2. In terms of medications use, most patients (56.1%) used a combination of ICS, long-acting beta2-agonists (LABA), and montelukast for their treatment, and the number of participants who used this combination in the UC group (74.4%) significantly differs from those in the PC group (60.5%) and the WC group (48.7%). Monotherapy with ICS or montelukast was mostly used by the WC group (21.4%).

To compare whether FeNO is different among the three groups of patients who had different asthma control status.  Figure 3 provides information that the median of FeNO in the UC group (50 ppb) is significantly higher than those in the PC group (25 ppb) and the WC group (21 ppb), *p* < 0.001. However, there is no significant difference in the medians of FeNO between the PC group and the WC group (*p*=0.446).

**Figure 4:**
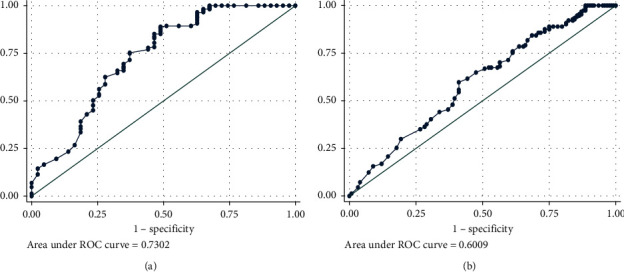
The area under the ROC curve of FeNO in detecting (a) GINA-defined uncontrolled asthma and (b) GINA-defined well-controlled asthma.

The values of FeNO to classify patients with asthma following GINA-defined control asthma status are shown in Figure 4. The area under the ROC curve (AUC) in prediction of GINA-defined UC asthma is 0.730 (95% CI: 0.637–0.823; *p* < 0.001, Figure 4(a)). With the cut-off point of FeNO >50 ppb, the maximum Youden index is 0.401 and FeNO levels could detect UC asthma with the sensitivity of 51% and the specificity of 89%, PPV 46%, NPV 91%, PLR 4.6, NLR 0.54, and accuracy 0.6. This cut-off point coincides with the high level of FeNO as per the ATS categorization (FeNO > 50 ppb) [10]. The AUC in prediction of GINA-defined WC asthma is 0.601 (95% CI: 0.534–0.668; *p* = 0.004, Figure 4(b)). With the cut-off point of FeNO <25 ppb, the maximum index of Youden is 0.186 and FeNO levels could detect WC asthma with the sensitivity of 59% and the specificity of 60%, PPV 64%, NPV 54%, PLR 1.5, NLR 0.7, and accuracy 0.6. Although this cut-off point has low predicted value, it also coincides with the ATS-defined low level of FeNO (FeNO <25 ppb) [10] The results indicate that although our suggested cut-off point of FeNO level detecting WC asthma is similar with the ATS classification, our suggested predicted valuesare still low.

**Figure 5:**
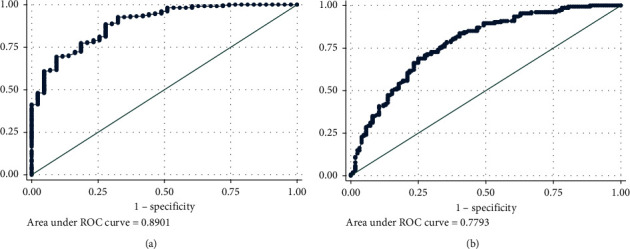
The area under the ROC curve of combination of FeNO and ACT in detecting (a) GINA-defined uncontrolled asthma and (b) GINA-defined well-controlled asthma.

In combination of FeNO and ACT, the AUC of this combination in detecting UC asthma and WC asthma was 0.890 (95% CI: 0.841–0.939; *p* < 0.001, Figure 5(a)) and 0.779 (95% CI: 0.716–0.827; *p* < 0.001, Figure 5(b)), respectively.

Using a combination of FeNO and ACT with usual cut-off points such as FeNO > 50 ppb and ACT < 20 points as an indicator for UC asthma, the value of this combination in predicting UC asthma is described in Table 4. From this table, the sensitivity, specificity, PPV, NPV, PLR, and NLR were calculated as 40%, 96%, 63%, 90%, 10, and 0.6, respectively. On the other hand, if using the cut-off point of the combination (FeNO < 25 ppb and ACT ≥ 20) as an indicator for WC asthma, the value of this combination in predicting WC asthma were sensitivity, specificity, PPV, NPV, PLR, and NLR as 49%, 84%, 79%, 57%, 3, and 0.6, respectively, calculated from the values given in Table 5.

## 4. Discussion

The main findings of the present study are that FeNO levels in Vietnamese adults with asthma were significantly different according to asthma control status during treatment, although with broad overlaps. Moreover, it showed that the combination of FeNO and ACT provides a better method in detecting UC and WC asthma although FeNO alone may detect UC and WC asthma with cut-off points similar with the high and low thresholds of FeNO levels as recommended by the ATS [10].

Asthma control may be formed by many components such as duration of disease, history of smoking, allergic problems, disease's severity, dose of treatment, and patients' adherence. All these factors are given in Tables 2 and 3 except adherence.

The information of adherence was not collected in detail in the present study; however, it is believed that almost all participants in this study follow their treatment strictly due to the following reasons. The study clinic is specialized in asthma and COPD management located in a teaching hospital; therefore, the model of its asthma management is standardized. There are 4 barrier layers which protect patients from incorrect usage of inhaler devices in this clinic: (i) giving general guidance on how to use the devices by doctors in examination room, (ii) guiding patients to use their real medications (after getting from hospital's pharmacy) by nurses at reception area, (iii) checking patients with their real performance with their old medications by spirometry technicians, whenever the patients were send to the spirometry room, and (iv) showing how to use medications by clips on TV in the waiting room. Furthermore, those patients who did not use their medications for more than 2 weeks within 3 months prior to the study were ruled out as mentioned in the exclusion criteria. With all of the above information, it is believed that adherence to treatment in this study is quite high and it may not distort the analyzed results.

Numerous studies have shown different FeNO levels among patients with asthma with different levels of asthma control defined by GINA, which is shown in Table 6. Nevertheless, the relationship between FeNO level and asthma control status may present in group of untreated patients but not in group of treated patients as reported by Visitsunthorn et al. [13]. Some authors believed that in patients on medium to high dose corticosteroid treatments, the FeNO level alone was not a good indicator for asthma control [6, 13]. Alvarez-Gutiérrez et al. [14] also indicated that an increased FeNO level could correspond to an UC asthma status, but that result was not statistically significant. In addition, FeNO may be useful in evaluating patients with eosinophilic airway inflammation but not in patients with neutrophilic or mixed-type airway inflammation [13–17]. Therefore, FeNO level in the later groups may not reflect asthma control status, which was also confirmed in several studies [13–17]. On the another hand, GINA-defined asthma control was based on patients' information over the preceding four weeks while FeNO measurement reflects inflammation on the day of assessment. Consequently, there could be a disagreement between the two evaluations, which has been observed in previous studies [14, 15, 17] but not in the present study. Because of this reason, it is suggested to use a combination of FeNO level with other measurements such as spirometry or questionnaire for a complete assessment for the asthma control status in patients with asthma since asthma is a heterogeneous disease [18, 19]. In the study of the combination of FeNO and questionnaire related to asthma control, Plaza et al. found that the addition of FeNO to ACQ-7 (asthma control questionnaire) increased the detectability of not-well-controlled asthma (compare to using ACQ-7 alone) [20]. In this study, the authors found that the combination of FeNO to ACQ-7 increased the detection of uncontrolled asthma by 14.8% following the maintenance therapy adjustment.

Only a few studies have been conducted to determine an appropriate cut-off point of FeNO level to predict a control status of asthma. Studies usually focus on detecting UC asthma rather than WC asthma because an UC status may lead to a bad prognosis. Early recognition of UC asthma may prevent adverse outcomes by adjusting treatment such as giving more educational intervention about the disease, correcting patients' inhaler technique, or stepping-up treatment following the current guidelines. The cut-off points found in these UC asthma detections ranged from 30 to 45 ppb with the sensitivity of about 67%, the specificity of 66%, and the positive predicted value of 85–88% [6, 25, 26]. In determining UC asthma, the cut-off point we found is FeNO level >50 ppb corresponding with the ATS-defined high level of FeNO with the specificity of 89%, which is much higher compared to other studies due to using a higher cut-off point [13, 18, 19]. This is understandable since a higher level of the cut-off point value could provide a lower false positive rate and a higher specificity of the test. If choosing the threshold of FeNO >50 ppb and ACT <20 as UC asthma criterion, this threshold can detect this status with the specificity of 96% and the PLR of 10. This high specificity and PLR means that the combination of FeNO and ACT has high value in recognizing uncontrolled asthma.

Identifying the cut-off point to detect WC asthma is more difficult because many studies found that FeNO levels ranged from 22 to 44 ppb in patients with asthma with a WC status [10]. In the present study, the value of FeNO alone or combination with ACT in detecting well-controlled asthma are not high (the AUC of FeNO alone was 0.60 and the AUC of combination was 0.78).

In summary, FeNO seems to be useful to predict WC and UC asthma in Vietnamese adult asthmatics who were on treatment for asthma. This biomarker can predict UC asthma with a cut-off point of >50 ppb and WC asthma with a cut-off point of <25 ppb. Additionally, the FeNO level may provide add in information to ACT as a surrogate for asthma control.

The present study has certain limitations. Various factors or comorbidities affect FENO measurements including eczema, atopy, allergic rhinitis, indoor air pollution, outdoor air pollution, and allergen exposure, which were not measured and adjusted for in the analysis. In addition, almost all patients in the current study were receiving ICS therapy, which is well known to affect the levels of FeNO and leading to a dose-dependent decrease, which was not adjusted in analysis. Moreover, the study population was limited in one hospital and was an observational study, which may not be representative for a general population of Vietnamese asthmatics. Another limitation is that the advantage of FeNO in asthma management recently is predicting the risk of exacerbation. However, this cross-sectional study did not investigate this issue.

## 5. Conclusions

Our present study showed that FeNO level was related to the levels of asthma control. The cut-off point to identify GINA-defined WC asthma and UC asthma were <25 ppb and >50 ppb, respectively. Those two cut-off points match with the categorization of ATS in low (25 ppb) and high (50 ppb) FeNO level. The combination of FeNO and ACT providedbetter information regarding asthma control than FeNO alone, and this combination is useful to predict asthma control statuses in Vietnamese asthmatic population.

## Figures and Tables

**Table 1:** Global Initiative for Asthma (GINA) definitions of asthma control.

Asthma symptom control		Level of asthma symptom control		
In the past 4 weeks, has the patient had		Well-controlled	Partly controlled	Uncontrolled
Daytime asthma symptoms more than twice/week?	Yes □ No □	None of these	1-2 of these	3-4 of these
Any night waking due to asthma?	Yes □ No □			
Reliever needed for symptoms^*∗*^ more than twice/week?	Yes □ No □			
Any activity limitation due to asthma?	Yes □ No □			

**Table 2:** Clinical characteristics of the study subjects according to asthma control status.

Characteristic	Total *n* = 278 (100%)	GINA 2017-defined asthma control, *n* (%)			*p*
		UC	PC	WC	
		43 (16)	81 (29)	154 (55)	
Age (years), mean (SD)	44 (13)	42 (14)	46 (13)	42 (13)	0.095
Female, *n* (%)	188 (68)	28 (65)	57 (70)	103 (67)	0.802
Weight (Kg), mean (SD)	57 (9)	59 (8)	58 (10)	57 (9)	0.292
Height (cm), mean (SD)	159 (7)	158 (8)	158 (8)	159 (7)	0.961
BMI (kg/m^2^), mean (SD)	23 (3)	24 (3)	23 (3)	23 (3)	0.112
Duration of asthma (years), mean (SD)	10 (15)	13 (17)	8 (12)	10 (15)	0.155
History of smoking, *n* (%)	23 (8)	7 (16)	4 (5)	12 (8)	0.088
Family history of allergy, *n* (%)	67 (24)	14 (33)	14 (17)	39 (25)	0.145
Personal history of allergy, *n* (%)	226 (81)	39 (91)	67 (83)	120 (78)	0.153

UC, uncontrolled; PC, partly controlled; WC, well-controlled; BMI, body mass index; SD, standard deviation; FeNO, fractional exhaled nitric oxide; FEV1, forced exhaled volume in 1 second; P, *p* value of comparison of 3 groups which had different levels of asthma control by the chi-square test; %, percentage counted by column.

**Table 3:** The distribution of patients in different levels of asthma control according to FEV1 % predicted, GINA treatment steps, and medication use.

Characteristic	Category	GINA 2017-defined asthma control, *n* (%)				*p*
		UC	PC	WC	Total	
%FEV1	<60%	7 (16.3)	6 (7.4)	2 (1.3)	15 (5.4)	<0.001
	60–79.9%	18 (41.9)	29 (35.8)	23 (14.9)	70 (25.2)	
	≥80%	18 (41.9)	46 (56.8)	129 (83.8)	193 (69.4)	
	Total	43 (100)	81 (100)	154 (100)	278 (100)	
GINA treatment steps	Step 2	5 (11.6)	7 (8.6)	50 (32.5)	62 (22.3)	<0.001
	Step 3	9 (20.9)	32 (39.5)	48 (31.2)	48 (31.2)	
	Step 4	29 (67.4)	42 (51.9)	127 (45.7)	127 (45.7)	
	Total	43 (100)	81 (100)	154 (100)	278 (100)	
Medication use	Montelukast	1 (2.3)	1 (1.2)	4 (2.6)	6 (2.2)	0.003
	ICS	2 (4.7)	2 (2.5)	29 (18.8)	33 (11.9)	
	ICS + montelukast	1 (2.3)	2 (2.5)	8 (5.2)	11 (4.0)	
	ICS + LABA	7 (16.3)	27 (33.3)	38 (24.7)	72 (25.9)	
	ICS + LABA + montelukast	32 (74.4)	49 (60.5)	75 (48.7)	156 (56.1)	
	Total	43 (100)	81 (100)	154 (100)	278 (100)	

UC, uncontrolled; PC, partly controlled; WC, well-controlled; FEV1, forced exhaled volume in 1 second; GINA, global initiative for asthma; ICS, inhaled corticosteroid; LABA, long-acting beta2-agonist; P, *p* value of comparison of 3 groups had different levels of asthma control by the chi-square test; %, percentage counted by column.

**Table 4:** Cross-table of combination of FeNO and ACT in detecting uncontrolled asthma.

		GINA control status		Total
		UC asthma	Controlled asthma^*∗*^	
FeNO + ACT	UC asthma (FeNO > 50 ppb and ACT < 20)	17	10	27
	Controlled asthma (FeNO ≤ 50 ppb or ACT ≥ 20)	26	225	251
	Total	43	235	278

Diagnostic values of combination of FeNO and ACT in detecting uncontrolled asthma calculated from this table are as follows: Sensitivity: 40%, specificity: 96%, PPV: 63%, NPV: 90%, PLR: 10, and NLR: 0.6. Chi-square test, *p* < 0.001. UC, uncontrolled; FeNO, fractional exhaled nitric oxide; ACT, asthma control test; PPV, positive predictive value; NPV, negative predictive value; PLR, positive likelihood ratio; NLR, negative likelihood ratio; ^*∗*^included GINA-defined partly controlled and well-controlled asthma.

**Table 5:** Cross-table of combination of FeNO and ACT in detecting well-controlled asthma.

		GINA control status		Total
		WC asthma	Not WC asthma^*∗*^	
FeNO + ACT	WC asthma (FeNO < 25 ppb and ACT ≥ 20)	75	20	95
	Not WC asthma (FeNO ≥ 50 ppb or ACT < 20)	79	104	183
	Total	154	124	278

Diagnostic values of combination of FeNO and ACT in detecting well-controlled asthma calculated from this table are as follows: Sensitivity: 49%, specificity: 84%, PPV: 79%, NPV: 57%, PLR: 3, and NLR: 0.6. Chi-square test, *p* < 0.001. WC, well-controlled; FeNO, fractional exhaled nitric oxide; ACT, asthma control test; PPV, positive predictive value; NPV, negative predictive value; PLR, positive likelihood ratio; NLR, negative likelihood ratio; ^*∗*^included GINA-defined partly controlled and uncontrolled asthma.

**Table 6:** FeNO values among groups with different GINA-defined asthma control levels in different studies.

Author, year	Sample size	Mean/median	WC asthma	PC asthma	UC asthma	Statistical method	*p*
Ferdaous et al., 2012 [21]	37	NA	29.1	48.2		NA	<0.05
Kim et al., 2015 [22]	155	Median	32.3	63.7		Mann–whitney	0.008
Visitsunthorn et al., 2013 [13]	20 (ICS naïve)	Median	31.8	34.1	92.0	Kruskal–wallis	<0.05
	94 (with ICS)	Median	19.2	24.9	39.2	Kruskal–wallis	0.24
Ricciardolo et al., 2016 [23]	363	Mean	NA	NA	42.90	NA (mean)	<0.001
Kamide et al., 2017 [24]	128	Mean	17.5	37.1	88.8	ANOVA	<0.005
Kavitha et al., 2017 [25]	151	Median	25.5	35	40	NA	<0.001
This study, 2017	278	Median	21.0	25.0	500	Kruskal–wallis	<0.001

UC, uncontrolled; PC, partly controlled; WC, well-controlled; mean or median of FeNO are shown; NA, not available.

## Data Availability

The data used to support the findings of this study are available from the corresponding author upon request.
